# Supplementary material for: AI-Enhanced Conversational Agents for Personalized Asthma Support in People With Asthma: Factors for Engagement, Value, and Efficacy in a Cross-Sectional Survey Study
Source: JMIR Hum Factors. 2026 Mar 11;13:e80979. doi: 10.2196/80979 (PMC12978652; doi:10.2196/80979)
Supplement: Multimedia Appendix 1 [file humanfactors-v13-e80979-s001.pdf]

Questions

1) Thank you and welcome. This brief survey asks about your experience of managing asthma. The insights you provide in this research will help improve technologies that support asthma management and risk assessment. The survey should take about 10 minutes to complete. Our institute will handle all data confidentially in accordance with the official College policies (see: https://bit.ly/2Vq9YjY). Participation is voluntary. You may withdraw at any time. No information will be gathered that might be used to identify you. The full document containing information on the project can be found here: https://drive.google.com/file/d/1PWRTODRub7dG-YyN2zyLNNnwJVdln-av/view?usp=sharingBy continuing, you indicate you agree to participate. If you don't wish to participate, use the back arrow at the top left of your screen to exit.

   Agree and continue.

2) How old are you?
   - 18-30
   - 31-40
   - 41-55
   - 55+
   - Prefer not to say.

3) Where do you spend most of your time?
   - In a major metropolitan area (big city)
   - In a small or medium-sized town
   - In a village or rural area

4) Do you identify as being part of a minority ethnic group, such as Black, Indigenous, Asian or mixed ethnicity?
   - Yes.
   - No.
   - Prefer not to say.

5) What is the highest level of education you have completed?
   - Did not attend school
   - Primary School
   - Secondary School or Sixth Form
   - University Degree
   - Trade/technical/vocational training

6) How much do you agree or disagree with the following statement: „I trust the UK healthcare system“
   - Agree
   - Somewhat Agree
   - Not sure
   - Somewhat Disagree
   - Disagree

7) Overall, how confident do you feel using computers, smartphones or other digital devices to do the things you need to do online?
   - Very confident
   - Somewhat confident
   - Only a little confident
   - Not at all confident

8) How often do you use messaging apps on a smartphone (such as WhatsApp, Microsoft Messenger, SnapChat etc.)?
   - Daily
   - A few times a week
   - A few times a month
   - Rarely or never

9) How often do you use WhatsApp specifically?
   - Daily
   - A few times a week
   - A few times a month
   - Rarely or never

10) Where do you normally look for information, tips or advice about asthma? (choose all that apply)
   - Internet search (e.g. “Google it“)
   - Asthma nurse
   - Doctor
   - Friends or family
   - Other

11) You chose “other“. Where else do you go for asthma information?

12) Have you ever used a mobile app for your asthma (for example, to learn about asthma or to track your symptoms)?
   - Yes.

- No.

13) Which asthma app have you tried?

14) Have you ever filled out an online questionnaire to check your asthma severity (for example, on the AsthmaUK website)?
    - Yes.
    - No.
15) Does your current mobile phone plan allow you to send unlimited text messages (without extra cost)?

    - Yes, I can send unlimited text messages.
    - No, I have a limit to the number of text messages I can send in a month.
    - I'm not sure.

16) Please describe what a "chatbot" is in your own words... (if you are unsure, just take a guess).

17) Please describe what a "virtual assistant" is in your own words... (if you are unsure, just take a guess.)

18) NOTE: For the rest of the survey, we'll use the term "virtual assistant" to mean: A non-human helper that you can chat with online via text. Examples of this include the Amazon Customer Support Chat shown in the image. Have you ever used a virtual assistant? (for example, to ask customer service questions on a website, to get help online or just for fun.)
    - Yes, definitely.
    - Yes, I think so, but I'm not completely sure.
    - No.

19) What did you use it for?

20) We are interested in your views on virtual assistants. How much would you agree with the following statements...

    Virtual assistants can be *useful*
    - Strongly Agree
    - Somewhat Agree
    - Neutral
    - Somewhat Disagree
    - Strongly Disagree

21) Virtual assistants are *not reliable*
- Strongly Agree
- Somewhat Agree
- Neutral
- Somewhat Disagree
- Strongly Disagree

22) Virtual assistants are can be*fun to use*
- Strongly Agree
- Somewhat Agree
- Neutral
- Somewhat Disagree
- Strongly Disagree

23) I'd always prefer to talk to a real person than a virtual assistant.
- Strongly Agree
- Somewhat Agree
- Neutral
- Somewhat Disagree
- Strongly Disagree

24) I'm happy to use a virtual assistant sometimes.
- Strongly Agree
- Somewhat Agree
- Neutral
- Somewhat Disagree
- Strongly Disagree

25) It's easier to use a virtual assistant than to make a phone call.
- Strongly Agree
- Somewhat Agree
- Neutral
- Somewhat Disagree
- Strongly Disagree

26) Virtual assistants can provide a faster response to my concern.
- Strongly Agree
- Somewhat Agree
- Neutral
- Somewhat Disagree
- Strongly Disagree

27) Imagine you could use your mobile phone to have a text chat with a virtual assistant called "AsthmaBot". AsthmaBot is a computer system that has knowledge about asthma. It can ask you questions about your symptoms and then give you personalised answers about how severe your asthma is. It can also predict how likely you are to have an asthma attack in the future, and help you come up with ideas for improving your asthma that work for your lifestyle. Imagine you can chat with AsthmaBot using a messaging service like WhatsApp. How interested would you be in trying out AsthmaBot?
   - Very interested
   - Somewhat interested
   - Neutral
   - Not particularly interested
   - Not at all interested

28) What would make you more likely to try it?

29) What concerns might you have about chatting with a virtual assistant about asthma risk?

30) How would you most prefer to chat with AsthmaBot? (You can choose more than one)
   - Through WhatsApp
   - Through a website
   - Through text messaging (SMS)
   - Using a custom app
   - Using a voice assistant like Alexa
   - No preference

31) Imagine AsthmaBot could detect your asthma severity by listening to the *sound of your voice*. You would speak into your phone to provide a short voice recording and AsthmaBot would analyse this recording for signals (that only a computer can detect) to determine your asthma severity. How interested would you be in trying this voice feature?
   - Very interested
   - Somewhat interested
   - Neutral
   - Not particularly interested
   - Not at all interested

32) Would the voice feature make you more or less interested in trying AsthmaBot?
   - The voice feature would make me more likely to try AsthmaBot.
   - The voice feature would make me less likely to try AsthmaBot.

- It would make no difference.

33) Imagine AsthmaBot told you your risk of having an asthma attack in the next two months was high. Which of the following would you be interested in? (tick all that apply). I'd be interested to...
    - Understand how AsthmaBot calculated my risk.
    - Understand how accurate or reliable the calculation is.
    - Get advice on how I can lower the risk.
    - Get advice on how to manage an asthma attack.
    - Others

34) What “other“ things would you be interested in?

35) Please select the most *useful time span* for getting an asthma attack prediction. „I'd like to know the likelihood of me getting an asthma attack in the next ...
    - 1 week
    - 1 month
    - 2 months
    - 3 months
    - 6 months
    - 12 months

36) Imagine AsthmaBot helped you to plan actions to lower your risk. For example, you decided to keep your pet out of the bedroom, and vacuum more often. Which of the following would you find helpful to do with AsthmaBot afterward? (Tick all that apply).
    - I'd like to tell AsthmaBot to occasionally remind me of the things I planned to do.
    - I'd like AsthmaBot to check in with my symptoms over time to help me keep my risk low.
    - I'd like to use AsthmaBot to check my risk occasionally to see what strategies are working.
    - I'd like to use AsthmaBot occasionally to see if my risk changes over time.
    - I'd only use AsthmaBot as a one-off.
    - Other.

37) What *other* things would you find helpful to do with AsthmaBot?

38) What kind of conversational style do you think AsthmaBot should use to communicate? (Tick all that apply). AsthmaBot should be...

- Friendly
- Light-hearted
- Humorous
- Informal
- Caring
- A good listener
- Serious
- Direct
- Authorative
- Like talking to a friend
- Like talking to a nurse
- Like talking to a robot
- Like talking to a doctor
- Reassuring
- Other

39) What *other* words would you use to describe how AsthmaBot should be?

40) What organisation would you trust most to create and provide a service like AsthmaBot?

- NHS
- An asthma charity
- A health insurance company
- A technology company
- No preference
- Other

41) What *other* organisation would you trust to provide the AsthmaBot service?

42) The last few questions about your asthma. I was diagnosed with asthma...

- When I was under 16.
- When I was over 16.
- Prefer not to say.

43) How long ago were you diagnosed with asthma?

- Within the last 6 months.
- More than 6 months ago.
- Prefer not to say.

44) How serious do you think your asthma is?
- Very serious
- Somewhat serious
- Not serious

45) How confident are you in dealing with your asthma to stop symptoms and attacks?
- Very confident
- Somewhat confident
- Not confident

46) In the last month have you had difficulty sleeping because of your asthma symptoms (including cough)?
- Yes.
- No.

47) In the last month, have you had your usual asthma symptoms during the day (cough, wheeze, chest tightness or breathlessness)?
- Yes.
- No.

48) In the last month, has your asthma interfered with your usual activities (housework, work, school, etc.)?
- Yes.
- No.

49) Have you visited hospital (A&E) for emergency treatment because of asthma in the past two years?
- Yes.
- No.

50) When managing your asthma, tick the boxes if you get support from:
- Family and friends
- My GP or nurse
- Other
