# Supplementary figures and images for: AI-Enhanced Conversational Agents for Personalized Asthma Support in People With Asthma: Factors for Engagement, Value, and Efficacy in a Cross-Sectional Survey Study
Source: JMIR Hum Factors. 2026 Mar 11;13:e80979. doi: 10.2196/80979 (PMC12978652; doi:10.2196/80979)

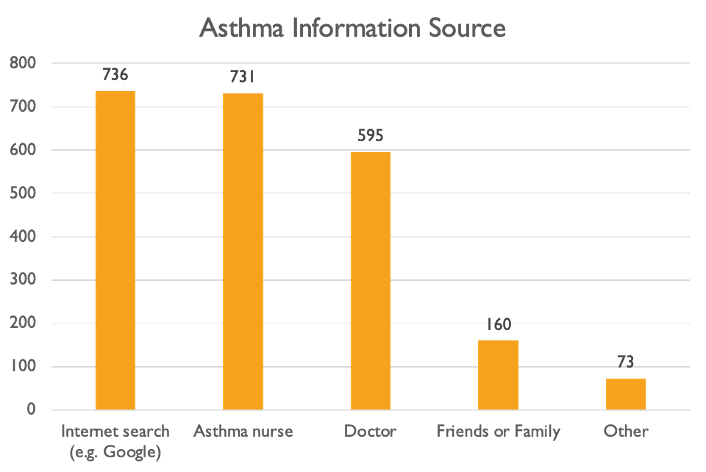

Supplement: Multimedia Appendix 2 [file humanfactors-v13-e80979-s002.png]

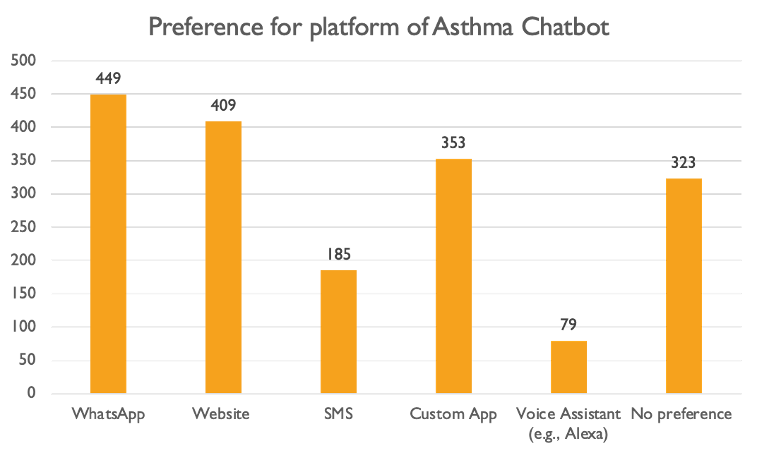

Supplement: Multimedia Appendix 3 [file humanfactors-v13-e80979-s003.png]
